# On the Employment of a New Remedy, the *Monesia*, in Menorrhagia, Diarrhœa, &c.

**Published:** 1840-09

**Authors:** 


A new article of the materia medica, the *Monesia*, of South American growth, having attracted no inconsiderable share of attention in the French metropolis, has been recently introduced to the profession in this country by Joseph G. Nancrede, M.D., whose opinion of the eclat which it is destined to win, may be inferred from his eagerness to be the first to extend a welcoming hand to the new comer. We introduce the stranger to our readers, by presenting Dr. Nancrede's letter.

*To the Editors of the Medical Examiner:*

Gentlemen:—With the intention of extending a knowledge of the properties of monesia, and of thus rendering practically useful, to our community, the very interesting paper of Dr. Martin Saint Ange, a translation of which appeared in the last number of your journal, I beg leave, through the medium of its pages, to acquaint the medical profession, that a portion of monesia, recently received from Paris, has been placed in the hands of Mr. F. Brown, corner of Fifth and Chesnut streets, where it can be obtained by those who may wish to prescribe it.

The preparations which have reached me, and which are to be obtained here, are, 1, the aqueous extract; 2, a syrup containing about six grains of the extract to the ounce; 3, a hydro-alcoholic tincture, containing about thirty-two grains to the ounce; 4, an ointment, containing one-eighth of its weight of extract.

While on this subject, I may be permitted to state, that having had in my possession for about a week, this new substance accompanied by the *Gazette Medicale de Paris*, of 19th

October, 1839, in which I read Dr. Martin Saint Ange's original paper, and also the *Notices sur le Monesia* by M. Bernard Derosne, to whom is due the credit of having introduced the new article to the notice of the profession, and who, in conjunction with M. O'Henry, has made of it a chemical analysis, I naturally felt desirous of being the first on this side of the Atlantic to test the properties of so interesting a remedy.

Accordingly I have exhibited it in five cases; and though it may be premature, in so short a period, to form any positive conclusions, yet I may be allowed to state, that, during the short period of its use in my hands, the medicine has realised the expectations held out by its friends in Paris.

I will merely add for the present, that I have exhibited the extract internally in the form of pills, in three cases, of which the first was diarrhœa, of long standing. Here its tonic and astringent qualities evidently arrested the disease. The two next cases were those of patients laboring under dysmenorrhœa; in both which, the symptoms have been materially improved under its exhibition. The fourth case is one of ulceration of the mouth, involving the tongue, of some week's duration, which I have treated by a solution of one part of the tincture, to five of water, as a local application, for the three last days, I think, with some benefit to my patient. The fifth case, is one of scrofulous ulceration, upon which I have applied the powdered extract twice. As yet, I cannot discover any change for the better.

These cases of the administration of the monesia, in several of its preparations, are recent, the oldest being only of a week's date, yet the results are so far encouraging, as to induce the wish that the American Medical Profession, which has now an opportunity of becoming acquanted with monesia, may fairly and fully test the properties of this new addition to the Materia Medica.

*Walnut street*, April 1840.

---

A translation of Dr. G. J. Martin St. Ange's paper was published in the London Gazette, and copied from thence into the Examiner; we cannot find room for it, but may say that it is a collection of the testimony of a number of eminent French practitioners in favor of the efficacy of monesia as a remedy for nearly all sorts of diseases:—diarrhœa, hæmoptysis, chronic coughs, scrofula, menorrhagia, dyspepsia, &c. &c.

In the Medical Examiner, of July 11th, 1840, a very interesting case of menorrhagia, of seven weeks' duration, cured by the extract of monesia, is reported by A. D. Chaloner, M. D., which is worthy of attentive perusal, and with it we shall close the testimony that has been adduced in favor of the new remedy.

On *Friday, July, 2d,* at 2½ o'clock, P. M., I was called to see Mrs. L——rs, æt. 35, of sanguine temperament, who was suffering under a severe attack of menorrhagia of *seven weeks' duration.* A month previous to the attack, she had miscarried, (three and a half months advanced in pregnancy,) and recovered under the usual course of treatment; but having over exerted her strength, and taken cold, she was attacked with a profuse "flow of blood from her womb," at first mistaken for the return of the catamenia. It increased, however, gradually, and she had recourse, previously to my visit, to various cold and astringent injections, in connexion with the elixir vitriol. These remedies failing, and the discharge increasing, she became alarmed and sent for me. I found her, on my arrival, sitting up in a chair, countenance pale and anxious, pulse slow, violent pains in lumbar and sacral region, and the slightest motion causing a profuse discharge. She was immediately placed in her bed, ordered to take *cold* drinks, and the following recipe: ℞. Acetat. Plumbi, ℈jss., G. Opii, gr. iv. M. ft. pil. No. xii. S. one every hour, and an injection per vaginam of ℞., Sulphat. Zinci, gr. viii., Aq. Font. f℥i.—her feet and legs were elevated by means of blocks of wood placed beneath the feet of the bedstead, and *perfect rest* enjoined.

*July 3d.* Had slept some, pulse more natural, skin cool, the discharge undiminished.

*Treatment.*—Increase the acetas plumbi, &c. to 8 grs. every hour, in conjunction with a third of a grain of opium; continue injections; ice in a bladder to be applied over the pubic region.

*Vesper.* Feels better; a perceptible diminution of the discharge; treatment to be continued as before.

*July 4th.* Having been prevented from seeing Mrs. L——rs to-day, my friend, Dr. N. Benedict, saw her for me, and finding the hæmorrhage returning, and bowels constipated, gave her the following ℞. Secale Cornutum, ʒi., Sacch. Albi., G. Acaciæ, q. s., Aq. Font. f℥vi., M. S. a table-spoonful every four hours,—also, these pills: ℞. Hieræ Picræ, ʒss., Sapo.

Venet. gr. iv., Syrup. Rhei, q. s., M. ft. pil. x., S. two every night.

*July 5th.* Bowels opened; discharge still continues, and profuse, and increased by motion; treatment continued.

*July 6th.* Returned to the city and saw her with Dr. B. No abatement of the discharge. Treatment—to take of the infusion of secale cornutum a table-spoonful every hour; elixir vitriol, 30 gtt. in " eau sucre," at the same time.

*Vesper.* Discharge continues; feels very weak. Treatment—℞. Secale Cornutum Pulv. Sacch. Alb. āā ℈ij, M. ft. pulv. No. viii., S. one every half hour until pains are produced.

*July 7th.* Has taken *seventy grains* of ergot without causing uterine pains, or affecting the discharge. Treatment—℞. Acet. Plumbi ℈jss., Tict. Opii fʒj., Aq. Font. f℥ij., ft. enema, S, one half at once, the remainder in an hour; elixir vitriol, as a tonic, iced drinks, astringent injections per vaginam, warming plaster to sacral region.

*July 8th.* The uterus, on examination per vaginam, was soft and spongy; in the commencement of the treatment it was slightly swollen, and the anterior lip enlarged; pain in the back and the discharge continue. Treatment—℞. Tinct. Cinnamon f℥ij., S. thirty drops every hour; ℞. Prussiat Ferri, ʒj., G. Aloes, gr. v., Conserv. Rosar. q. s., M. et divid. in pil. xx. S. one four times daily; alum water injections, a compress of folded napkins, and a broad roller, applied tightly over the uterine region.

*Vesper.* Feels better; bowels opened freely; to take but two pills and tinct. cinnamon, as before; discharge as before.

*July 9th.* The discharge having increased in frequency and quantity, I procured twelve pills of the EXTRACT OF MONESIA, of three grains each, and at 2 o'clock, P. M. gave her six pills—*one* to be taken every *hour and a half*, until they had an effect upon the discharge.

*Vesper.* Has taken about three pills, (3 grs. each ;) the first pill having caused *pretty severe uterine pains*—as she expressed herself, "*as if she was going to be sick*," (i. e. be confined ;) after taking the third pill, the discharge was "*a mere show.*" Treatment—to take the remaing three pills, one every two hours.

*July* 10. Slept well ; pain in the back gone ; *the discharge entirely ceased;* feels well ; pulse natural ; appetite good ; skin cool; the uterus contracted and free from pain, on examination per vaginam. Treatment—perfect rest, nourishing diet and cold drinks.

*Vesper.* Improving; no return of the discharge.

*July* 11. Still *no return* of the hemorrhage; gaining strength; to take tonics, &c.

*July* 12. Appetite indifferent; *no return.* ℞. Pulv. Colombœ, Pulv. Sub-Carb. Ferri, Rhei, Zingiberis aa ʒj. M. ft. chart. No. xii. S. one three times a day in molasses. Infusion of Prunus Virginiana, a wine-glassful three times a day. Diet—oysters, &c.

*Vesper.* Has had, owing to the exertion required in changing her bedding, some slight discharge; checked by one or two pills of the *monesia,* every four hours.

*July* 16. Still using tonics; no pain; *no return of the hemorrhage;* feels well; anxious to get out of bed.

Supposing that the above case may be interesting as showing, that in the *Extract of Monesia* we have a substance capable of causing powerful uterine contractions, even in small doses, and of arresting profuse menorrhagia of long duration, after the exhibition of all the usual remedies had failed, even when pushed to the extreme; and trusting that this hasty outline of the *effects of monesia* in this, to me, troublesome (although not uninteresting) case, may be of some interest to members of the profession generally,

I remain, gentlemen,
Your obedient servant,
A. D. CHALONER, M.D.

*Philadelphia,* July 10, 1840.

In this case, the *monesia* appeared to arrest the hemorrhage by exciting contraction in the muscular fibres of the uterus, in consequence of which its tissue was condensed, and compression made upon the bleeding vessels. Muscular atony is a frequent cause of metrorrhagia, occurring after abortion or labor at the full term, and in such cases we have been long accustomed to prescribe the *ergot,* in pills or emulson, with the most satisfactory results; indeed, in our hands, it so uniformily succeeds that we are not a little surprised at its failure in Dr. Chaloner's case. It is scarcely necessary to add that the sugar of lead, though a remedy of decided efficacy, when hemorrhage arises from a morbid state of the exhalent vessels, cannot possibly avail when impaired muscular contractility permits the blood to escape from their patulous mouths. On the contrary, it is probable that the lead, if administered in sufficient quantity, might *paralize* the uterine muscular fibres, as it is known to do certain of the voluntary muscles, and thus aggravate the discharge. Be this as it may, it is certain that lead is not to be confided in as a remedy for this special hemorrhage, and that ergot is calculated directly to fulfil the indication which it presents. If it should be found, on further trial, that the monesia belongs to the same class of remedies as the ergot and acts with greater certainty and efficiency in exciting uterine contraction, it will prove a valuable addition to the materia medica.

